# Evidence for capacity sharing when stopping

**DOI:** 10.1016/j.cognition.2015.05.014

**Published:** 2015-05-28

**Authors:** Frederick Verbruggen, Gordon D. Logan

**Affiliations:** a University of Exeter, UK; b Vanderbilt University, United States

**Keywords:** Response inhibition, Selective stopping, Dual tasking, PRP, Capacity sharing

## Abstract

Research on multitasking indicates that central processing capacity is limited, resulting in a performance decrement when central processes overlap in time. A notable exception seems to be stopping responses. The main theoretical and computational accounts of stop performance assume that going and stopping do not share processing capacity. This independence assumption has been supported by many behavioral studies and by studies modeling the processes underlying going and stopping. However, almost all previous investigations of capacity sharing between stopping and going have manipulated the difficulty of the go task while keeping the stop task simple. In the present study, we held the difficulty of the go task constant and manipulated the difficulty of the stop task. We report the results of four experiments in which subjects performed a selective stop–change task, which required them to stop and change a go response if a valid signal occurred, but to execute the go response if invalid signals occurred. In the consistent-mapping condition, the valid signal stayed the same throughout the whole experiment; in the varied-mapping condition, the valid signal changed regularly, so the demands on the rule-based system remained high. We found strong dependence between stopping and going, especially in the varied-mapping condition. We propose that in selective stop tasks, the decision to stop or not will share processing capacity with the go task. This idea can account for performance differences between groups, subjects, and conditions. We discuss implications for the wider stop-signal and dual-task literature.

## 1. Introduction

Stopping prepared but no longer relevant responses is a simple act of executive control that supports flexible and goal-directed behavior (Aron, Robbins, & Poldrack, 2014; Logan, 1994; Ridderinkhof, van den Wildenberg, Segalowitz, & Carter, 2004; Verbruggen & Logan, 2008c). In the last two decades, response inhibition has received much attention across research domains. Cognitive psychologists and neuroscientists have explored the cognitive and neural mechanisms of response inhibition, developmental scientists have studied the ‘rise and fall’ of inhibitory control capacities across the life span, and clinical psychologists, neuropsychologists, and psychiatrists have examined correlations between individual differences in response inhibition and behaviors such as substance abuse, overeating, pathological gambling, and risk taking (for reviews, see Aron et al., 2014; Bari & Robbins, 2013; Chambers, Garavan, & Bellgrove, 2009; Logan, 1994; Verbruggen & Logan, 2008c). Research on response inhibition has thus become a central component of the study of self-regulation and behavioral change (see e.g. Hofmann, Schmeichel, & Baddeley, 2012).

Most response inhibition studies implicitly or explicitly assume that stop processing occurs independently from go processing for most of the time. By making this assumption, the covert latency of the stop process can be estimated. Here we report the results of four experiments that used a selective stop–change task in which different signals could be presented; subjects were instructed to stop and change the planned go response if one of the signals occurred (valid signal), but to execute the planned go response if the other signals occurred (invalid signals). Our experiments challenge the dominant independent race model of response inhibition because they indicate that the processes underlying going and stopping can interact substantially, especially when the stop-signal rules change frequently. Our results also shed a new light on strategy selection in selective stop tasks.

### 1.1. A brief introduction to independent race models of inhibitory control

Reactive inhibitory control in response to changes in the environment or internal state is often studied in tasks such as the go/no-go task (Donders, 1868/1969) and the stop-signal task (Lappin & Eriksen, 1966; Logan & Cowan, 1984; Vince, 1948). In the go/no-go task, subjects are instructed to respond when a go stimulus appears (e.g. an ‘O’), but to withhold their response when a no-go stimulus appears (e.g. an ‘X’). In the stop-signal task, subjects perform a primary go task, such as responding to the identity of a stimulus (e.g. press left when an ‘O’ appears, and right when an ‘X’ appears). On a minority of the trials, an extra visual or auditory signal appears after a variable delay, instructing subjects to withhold the planned go response.

Performance in these tasks and their many variants can be modeled as an independent race between a go process, triggered by the presentation of a go stimulus, and a stop process, triggered by the presentation of the no-go stimulus or the stop signal (Logan & Cowan, 1984; Logan, Cowan, & Davis, 1984; Logan, Van Zandt, Verbruggen, & Wagenmakers, 2014; for a review, see Verbruggen & Logan, 2009a). When the stop process finishes before the go process, response inhibition is successful and no response is emitted (signal-inhibit); when the go process finishes before the stop process, response inhibition is unsuccessful and the response is incorrectly emitted (signal–respond). In the go/no-go task, the main dependent variable is the probability of responding on no-go trials. In the stop-signal task, the covert latency of the stop process (stop-signal reaction time or SSRT) can also be estimated from the independent race model (Logan, 1981; Logan & Cowan, 1984; Logan et al., 2014); this has made it a very popular paradigm for the study of response inhibition in cognitive psychology, cognitive neuroscience, developmental psychology, and psychopathology (Verbruggen, Chambers, & Logan, 2013; Verbruggen & Logan, 2008c).

The independent race model assumes independence between the finishing times of the go process and the stop process (Logan & Cowan, 1984). The independence assumption takes two forms: context independence (also referred to as signal independence) and stochastic independence. Context independence means that the go reaction time (RT) distribution is not affected by the presentation of stop signals. Stochastic independence means that trial-by-trial variability in go RT is unrelated to trial-by-trial variability in SSRT (in other words, the durations of the go processes and the stop processes are not correlated). These assumptions should not be taken lightly because SSRT cannot be reliably estimated when they are violated (Band, van der Molen, & Logan, 2003; Colonius, 1990; De Jong, Coles, Logan, & Gratton, 1990).

**Fig. 1:**
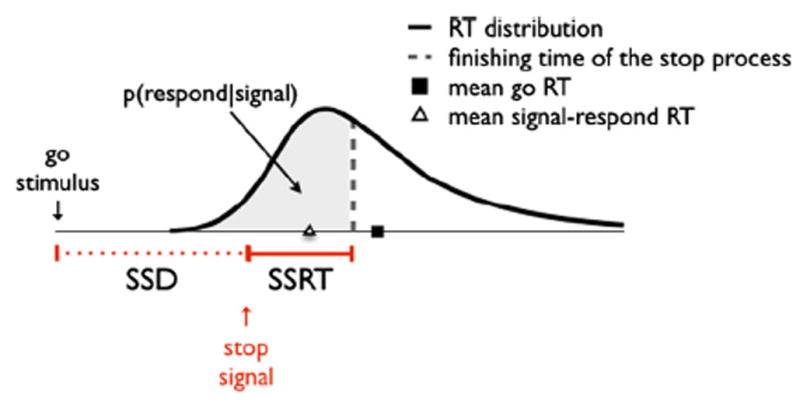
A graphic representation of the assumptions of the independent horse-race model of Logan and Cowan (1984)signal–respond trials, the go process finishes before the stop process. The gray area under the curve indicates the probability of a signal–respond trial. This figure shows why mean reaction time on signal–respond trials is shorter than mean RT on no-signal trials: the former is calculated based on the fastest RTs that escaped inhibition (i.e. the RTs on the left of the vertical dashed line), whereas the latter is calculated based on the whole RT distribution (i.e. the RTs on the left and right of the vertical dashed line). SSD = stop-signal delay; SSRT = stop-signal reaction time.

The independence assumptions can be tested by comparing the mean RT for signal–respond trials with the mean RT for no-signal trials, and by comparing RT distributions for signal–respond and no-signal trials (Verbruggen & Logan, 2009a). First, the independent horse-race model predicts that mean no-signal RT should be longer than mean signal–respond RT: mean signal–respond RT only represents the mean of those responses that were fast enough to finish before the stop signal, whereas mean no-signal RT represents the mean of all go responses (Fig. 1). Second, the independent race model predicts that signal–respond and no-signal distributions have a common minimum, but later diverge (Osman, Kornblum, & Meyer, 1986). A review of the literature revealed that the independence assumptions are met in most stop-signal studies (Verbruggen & Logan, 2009a). This conclusion is further supported by behavioral studies that directly tested dependence between going and stopping (e.g. Logan & Burkell, 1986; Logan et al., 2014; Yamaguchi, Logan, & Bissett, 2012), and by studies that modeled the processes underlying going and stopping (e.g. Boucher, Palmeri, Logan, & Schall, 2007; Logan, Yamaguchi, Schall, & Palmeri, 2015; Logan et al., 2014).

### 1.2. The interaction between going and stopping in stop–change and selective stop tasks

The independent race model provides a simple and elegant description of stop performance in go/no-go and simple stop-signal tasks, and it allows the estimation of the stopping latencies. It has also been applied to the stop–change task and the selective stop task to study cognitive flexibility and selectivity of action control in healthy and clinical populations and under various experimental conditions.

In stop–change tasks, subjects are instructed to stop the originally planned go response and execute an alternative ‘change’ response when a signal occurs (for reviews, see Boecker, Gauggel, & Drueke, 2013; Logan & Burkell, 1986; Verbruggen & Logan, 2009a). Experimental, computational, and neuro-imaging work suggests that subjects first inhibit the original go response (go1) and then execute the alternative ‘change’ response (Boecker et al., 2013; Camalier et al., 2007; Jha et al., 2015; Verbruggen & Logan, 2009a; Verbruggen, Schneider, & Logan, 2008). For example, in a previous study (Verbruggen, Schneider, et al., 2008), we manipulated the delay between the stop signal and a signal indicating which change response had to be executed (go2). As this delay increased, the probability of stopping the primary task response changed very little, which indicates that the stop process was not influenced by the go2 process. This supports the independence assumption of the race model (see also Logan & Burkell, 1986, who showed that stopping was not influenced by go1 processing). However, the latencies of the change response decreased substantially when the delay between the stop signal and the change signal increased (Verbruggen, Schneider, et al., 2008). We proposed that these findings were consistent with a serial model (i.e. the go1 response is canceled by a stop response, followed by the preparation and execution of the go2 response) or a limited-capacity parallel model with a capacity-sharing proportion that resembles serial processing (i.e. stopping is prioritized, so the selection and execution of the go2 response only starts properly once the stop process has finished).

In selective stop tasks, subjects are instructed to stop their response on some signal trials, but not on others (for a short review, see Bissett & Logan, 2014). There are two variants of the selective stop task: in stimulus selective stop tasks, different signals can be presented and subjects must stop if one of them occurs (valid signal), but not if the others occur (invalid signals); in motor selective stop tasks, subjects must stop some of their responses (critical responses) but not others (non-critical responses). Most researchers assume that the decision to stop or not does not interact with ongoing go processes, as it allows them to estimate the stopping latency. However, Bissett and Logan (2014) found that signal–respond RT and invalid-signal RT were sometimes longer than no-signal RT in stimulus-selective stop tasks. This suggests that selecting the appropriate response to the signal may interact with ongoing go processes (violating the context independence assumption of the independence race model; see above). A similar pattern of results was observed by De Jong, Coles, and Logan (1995) in a motor variant of the selective stop task: signal–respond RTs for critical responses and signal RTs for non-critical responses were longer than no-signal RT. This suggests violations of the independence assumptions. By contrast, in their simple stop task and a stop–change task, signal–respond RT was shorter than no-signal RT (De Jong et al., 1995), which is consistent with the context independence assumption of the independent race model.

In sum, going in the primary task and stopping are independent in stop–change tasks, whereas dependence between go and stop has been observed in some selective stop tasks (e.g. Bissett & Logan, 2014; De Jong et al., 1995). The go and stop process may interact when subjects have to decide whether they need to stop or not. The present study tested independence assumptions by manipulating the difficulty of selective stop tasks. If we were to find consistent violations of the independence assumption, this would have serious repercussions for the application of the independent race model to such tasks and for the wider response-inhibition literature.

### 1.3. The present study

In four experiments, subjects performed a primary go task, such as responding to a digit or letter. On some trials, a signal could appear on the left or right of the go stimulus. When the signal was valid, subjects had to stop their planned response and respond to the location of the signal instead. Invalid signals had to be ignored. We used a stop–change task because it could provide us with two measures of ‘reactive’ action control on valid signal trials: the latency of the stop response (SSRT) and the latency of the change response. SSRT can only be estimated when the assumptions of the race model are met, whereas the latency of the change response is measured directly. In other words, we were guaranteed an index of reactive action control even when the assumptions of the independence race model are violated (for an alternative procedure that provides an index of action control when the independence assumptions are violated, see e.g. Morein-Zamir, Chua, Franks, Nagelkerke, & Kingstone, 2006; Morein-Zamir & Meiran, 2003).

To manipulate difficulty in the stop task, we changed the signal rules that determined whether subjects had to stop–change or not. In each experiment, there were two groups: a varied-mapping group and a consistent-mapping group. In the varied-mapping group, the valid signal changed every four trials (Experiments 1–2) or every trial (Experiments 3–4). Consequently, subjects could not practice the valid-signal rule and the demands on the rule-based system remained high throughout the whole experiment. We predicted that this would lead to strong dependence between going and stopping. By contrast, in the consistent-mapping group, the valid signal remained the same throughout the whole experiment. We predicted that this would reduce dependency between go and stop: when strong associations between the stimulus and a single response are formed (in this case, the stop–change response), the appropriate response to the signal can be activated whilst rule-based (or algorithmic) processing is taking place in other tasks (cf. Kahneman, 2003; Logan, 1979, 1988; Schneider & Shiffrin, 1977; Shiffrin & Schneider, 1977). Thus, stop processing can occur independently from go processing for most of the time in the consistent-mapping group.

## 2. Experiments

**Fig. 2:**
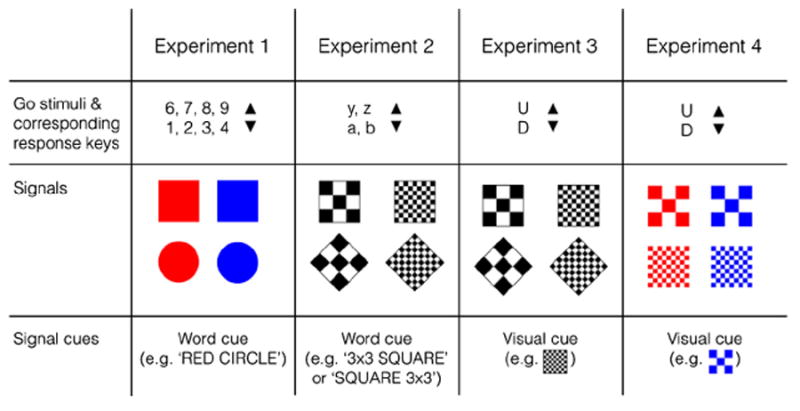
Overview of the go stimuli, corresponding response keys, change signals, and signal cues for each experiment. See Section 2.1 for further details.

In Experiment 1, the primary go task was a number magnitude task in which subjects had to decide whether a digit (the go stimulus) was smaller or larger than 5. On 25% of the trials (signal trials), a colored circle or square (i.e. the change signal; Fig. 2) could appear on the left or right of the digit. When the signal was valid (25% of the signal trials), subjects had to cancel their response to the digit and respond to the location of the signal instead. When the change signal was invalid, subjects had to ignore it and execute the go response as planned. At the beginning of each trial, we presented a word cue to indicate signal validity (e.g. ‘RED CIRCLE’). Subjects had to stop and change their response when the colored shape (i.e. the signal) matched the word cue. In the consistent-mapping group, the valid signal remained the same throughout the experiment, but in the varied-mapping group, it changed every four trials.

Experiment 2 was primarily designed to replicate the findings of Experiment 1 with different signals, different cues, and another primary task. The signals were 3 × 3 or 9 × 9 white-and-black chequerboards that could be rotated (square vs. diamond; Fig. 2). We presented a word cue to indicate signal validity at the beginning of each trial (e.g. ‘3 × 3 diamond’), but we counterbalanced the order of the cued features^1^ (Fig. 2). In the consistent-mapping group, the valid signal remained the same throughout the experiment, but in the varied-mapping group, it changed every four trials. In the primary go task, subjects decided whether a letter (a, b, y, or z; the go stimuli) occurred at the beginning or end of the alphabet. We used letters instead of digits to avoid overlap between the go stimulus and the cue.

In Experiment 3, we reduced memory demands but increased switch demands. Accuracy on no-signal trials was quite low for some subjects in Experiment 2 (see below), so we simplified the primary go task in Experiment 3: subjects had to decide whether the letter (the go stimulus) was ‘U’ or ‘D’. Each trial started with the presentation of a chequerboard (the cue) in the center of the screen. The cue was followed by the go stimulus (i.e. the letter). On signal trials, another chequerboard (the signal) appeared to the left or right of the go stimulus after a variable delay. Subjects were instructed to stop and change their response when the second chequerboard (i.e. the signal) matched the first chequerboard (i.e. the cue); on mismatch trials, subjects had to ignore the signal and execute the go response as planned. We expected that presenting the valid chequerboard at the beginning of each trial would reduce memory demands. However, we expected switch demands to increase because the valid signal could change on every trial in the varied-mapping group. Finally, Experiment 4 was primarily designed to replicate the findings of Experiment 3. In this experiment, we used colored chequerboards.

Initial analyses revealed that the difference between signal–respond RTs and no-signal RTs was (at least numerically) larger in the consistent-mapping groups than in the varied-mapping groups of all experiments. Furthermore, in each experiment we found that the varied-mapping group was more distracted by invalid signals (compared with no-signal trials) than the consistent-mapping group (as revealed by analyses of go accuracy, go RTs, or both). Finally, the analyses of performance for each individual also revealed strong similarities between experiments (Fig. 4). Therefore, we analyzed the data of all experiments together (total *N* = 192). This ensured that we had sufficient power (.80) to detect at least medium-sized effects in the (consistent-mapping vs. varied-mapping) between-groups comparisons.

### 2.1. Method

#### 2.1.1. Subjects

192 volunteers (48 per experiment) from the University of Exeter participated for monetary compensation (£5) or partial course credit. Nine subjects were replaced because their percentage of correct valid-signal trials was ≤20% (two in Experiment 1; three in Experiment 2; two in Experiment 3; and two in Experiment 4); two subjects in Experiment 2 were replaced because their percentage of correct no-signal trials was ≤80%; and one subject in Experiment 3 was replaced because of technical issues. All experiments of the present study were approved by the local research ethics committee at the School of Psychology, University of Exeter. Written informed consent was obtained after the nature and possible consequences of the studies were explained. The target sample and subject exclusion criteria were determined before data collection (based on a pilot study (*N* = 24) in which we found large effects of signal presentation in a consistent-mapping group).

#### 2.1.2. Apparatus, stimuli and procedure Experiment 1

The experiment was run on a 21.5-inch iMac using Psychtoolbox (Brainard, 1997). The change-signal cues were the words ‘RED SQUARE’, ‘BLUE SQUARE’, ‘RED CIRCLE’, and ‘BLUE CIRCLE’ (size: approximately 25 × 4 mm). The go stimuli were the digits 1–9 (excluding 5; stimulus size: approximately 2 × 4 mm). The word cues and go stimuli were centrally presented in a white font (Courier 20 point) on a black background. On signal trials, a visual signal appeared 200 pixels (approximately 4.5 cm) on the left or right of the go stimulus after a variable delay. There were four different signals (Fig. 2), which varied along two dimensions: color (red or blue; RGB = 255 0 0 and RGB = 0 0 255, respectively) and shape (square or circle; size: 7 × 7 mm). The signals occurred with equal probability. Subjects responded to the go stimuli (i.e. the digits) by pressing the ‘up’ (digit > 5) and ‘down’ (digit < 5) arrow key of a standard Mac keyboard with their right middle finger. They responded to the location of the signal (i.e. the colored shape) by pressing the left (signal = left) or right (signal = right) arrow key with their right index or ring finger, respectively.

There were two groups: In the consistent-mapping group, the valid signal remained the same throughout the whole experiment (but the signal-validity mapping was counterbalanced across subjects). In the varied-mapping group, the valid signal changed every four trials.

All trials started with the presentation of a signal cue (i.e. the words), indicating the valid signal (Fig. 2). The go stimulus (the digit) replaced the cue after 750 ms. Subjects had to decide whether the digit was smaller or larger than 5. The digit remained on the screen for 1,500 ms, regardless of RT. On 25% of the trials (signal trials), a signal was presented on the left or right of the digit after a variable delay. The location of the signal was randomized. When the signal matched the word cue (valid-signal trials; e.g. a red circle appeared when the cue was ‘RED CIRCLE’), subjects had to withhold the go (up/down) response and respond to the location of the signal instead (left/right). When the signal was invalid (invalid-signal trials; e.g. a red square occurred when the cue was ‘BLUE CIRCLE’), subjects had to ignore it and execute the go (up/down) response. There were 4 possible signals (Fig. 2). They occurred with equal probability, so only 25% of the signal trials (or 6.25% of all trials) were valid-signal trials. Valid and invalid signals were presented after a variable delay (change-signal delay; CSD). The CSD was initially set at 250 ms and continuously adjusted according to a tracking procedure to obtain a probability of successful change performance of .50. Each time a subject responded to the go stimulus or failed to execute the change response on a valid-signal trial, CSD decreased by 50 ms. When subjects successfully replaced the go response on a valid-signal trial, CSD increased by 50 ms. Subjects were informed about this tracking procedure and they were told not to wait for a change signal to occur. CSD for invalid-signal trials was yoked to the CSD for the valid-signal trials.

At the end of the trial, we presented feedback (on no-signal and invalid-signal trials: ‘correct’, ‘incorrect’, ‘not quick enough’ in case subjects did not respond before the end of the trial, or ‘no second response required’ in case they executed two responses; on valid-signal trials: ‘correct’, ‘try to stop response to digit’ in case they executed the go response, or ‘you must respond to signal’ in case they stopped the go response but did not execute the change response). The feedback remained on the screen for 500 ms, and the next trial started after a further 250 ms.

The experiment consisted of 12 blocks of 64 trials (768 trials in total, 48 of which were valid-signal trials). Subjects received a break after every block. During the break, we presented as feedback to the subjects their mean RT on no-signal trials, number of no-signal errors, number of missed no-signal responses, and percentage of correctly replaced responses. Subjects had to pause for 15 s.

#### 2.1.3. Apparatus, stimuli and procedure Experiment 2

These were the same as in Experiment 1 except for the following: There were four different signals (chequer-boards; Fig. 2; size: 12 × 12 mm), which varied along two dimensions: frequency (the number of squares inside the board; 3 × 3 or 9 × 9), and rotation (0° or 45°; square or diamond, respectively). The signals appeared approximately 4 cm on the left or right of the go stimulus after a variable delay. For half of the subjects, the word cues were ‘3 × 3 SQUARE’, ‘9 × 9 SQUARE’, ‘3 × 3 DIAMOND’, and ‘9 × 9 DIAMOND’; for the others, the cues were ‘SQUARE 3 × 3 ’, ‘SQUARE 9 × 9’, ‘DIAMOND 3 × 3 ’, and ‘DIAMOND 9 × 9’. The go stimuli were the letters ‘a’, ‘b’, ‘y’, ‘z’. Subjects responded to them using the ‘up’ (for the letters at the end of the alphabet) and ‘down’ (for the letters at the beginning of the alphabet) arrow keys. We used letters instead of digits to avoid interference between the go stimulus and the signal cue (which contained 2 digits).

#### 2.1.4. Apparatus, stimuli, and procedure Experiment 3

These were the same as in Experiment 2 except for the following: we showed the currently valid signal (a chequerboard) at the beginning of each trial (see above and Fig. 2). In the varied-mapping condition, the valid signal changed on every trial. The go stimuli were the letters ‘U’ and ‘D’, and subjects responded to them using the ‘up’ (U) and ‘down’ (D) arrow keys. Due to the randomization procedure, this experiment consisted of 3 blocks of 256 trials (768 trials in total). Subjects received a break after 64 trials. During the break, we presented as feedback on their performance for the last 64 trials.

#### 2.1.5. Apparatus, stimuli, and procedure Experiment 4

These were the same as in Experiment 3 except for the following: There were four different signals (chequerboards; Fig. 2), which varied along two dimensions: frequency or the number of squares inside the board (3 × 3 or 9 × 9), and color (red or blue; RGB = 255 0 0 and RGB = 0 0 255, respectively).

### 2.2. Analyses

All data processing and analyses were completed using R (R Development Core Team, 2014). All data files and R scripts used for the analyses are deposited on the Open Research Exeter data repository (http://hdl.handle.net/10871/17242).

Descriptive statistics for no-signal and invalid signal trials appear in Table 1; descriptive statistics for valid-signal trials appear in Table 2. Inferential statistics appear in Tables 3 and 4. Consistent with our previous research (Verbruggen & Logan, 2009b), we distinguished between the proportion of correct no-signal or invalid-signal trials and the proportion of missed no-signal or invalid-signal trials. However, probability of a missed go response was generally very low (mean: 0.016, *sd* = 0.021), and therefore not further analyzed. As discussed below, the independence assumptions of the race model were violated, especially in the varied-mapping group. Therefore, we did not estimate SSRT.

### 2.3. Results

**Fig. 3:**
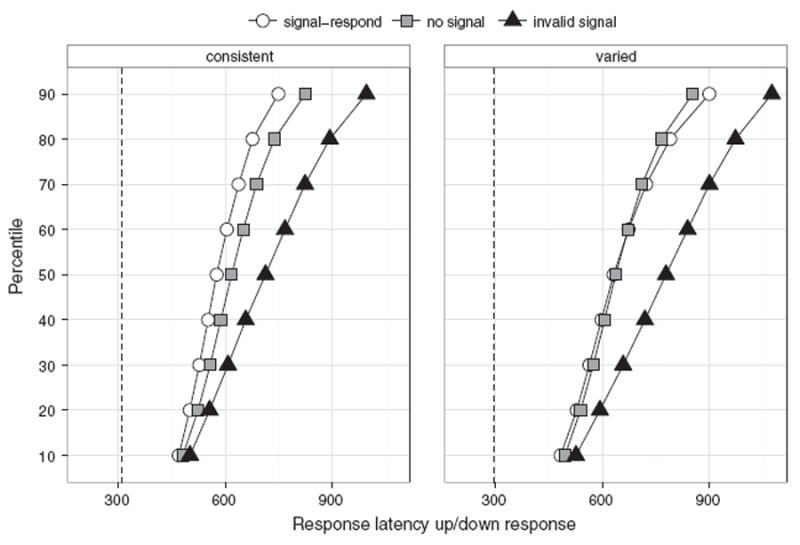
Quantile averages for signal–respond trials, no-signal trials, and invalid-signal trials for each group. For this graph, we included incorrectly executed go responses – e.g. when subjects pressed the ‘up’ key instead of the ‘down’ key. The dashed vertical lines indicate when valid signals were presented (on average).

We focused on go performance performance on no-signal, invalid-signal, and valid-signal trials to explore dependence between going and stopping. For each group, we calculated means (Tables 1 and 2) and plotted quantile averages for the different trial types (Fig. 3).

#### 2.3.1. Signal–respond minus no-signal RT

The independent horse-race model predicts that mean no-signal RT should be longer than mean signal–respond RT, and that signal–respond and no-signal distributions should have a common minimum, but later diverge with the signal–respond distribution to the left of the no-signal distribution (Osman et al., 1986; Verbruggen & Logan, 2009a). Here we tested both predictions.

We compared mean RT on signal–respond trials with mean RT on no-signal trials using a mixed ANOVA with Group and Experiment as between-subjects factors and Trial Type as within-subjects factor (Table 3). For this analysis, we included incorrectly executed go responses (e.g. when subjects pressed ‘up’ instead of ‘down’) because signal–respond RTs are usually the fastest RTs (so incorrect go responses are more likely to occur). Consistent with the context-independence assumption of the independent race model, mean signal–respond RT was shorter than no-signal RT in the consistent-mapping groups (global difference: −43 ms). However, in the varied-mapping group, signal–respond RT tended to be longer than no-signal RT (global difference: +7 ms). This difference between groups was reliable (Trial Type by Group: *p* < .001). No other interactions were statistically significant (Table 3).

These findings suggest dependence between go and stop in the varied-mapping group, but not in the consistent-mapping group. This conclusion was further supported by the comparison of the signal–respond and no-signal RT distributions (Fig. 3). In the consistent-mapping group, signal–respond RTs were consistently shorter than no-signal RTs (in other words, the signal–respond distribution was to the *left* of the no-signal distribution). In the varied-mapping group, signal–respond RT was longer than no-signal RT for the 70–90 percentiles (in other words, the signal–respond distribution was to the *right* of the no-signal distribution).

#### 2.3.2. Invalid-signal vs. no-signal trials

If the decision about the signal does not interfere with ongoing go processes (as most selective stop task users explicitly or implicitly assume), go performance should be similar for invalid-signal and no-signal trials. To test this prediction, we compared go RTs and probability of a correct go response [*p*(correct)] for invalid-signal trials and no-signal trials using a mixed ANOVA with Group and Experiment as between-subjects factors and Trial Type as within-subjects factor (Tables 1 and 4).

For the mean RT analysis, we included only trials on which the go response was correct. Mean go RTs were generally longer on invalid-signal trials^2^ than on no-signal trials (Trial Type: *p* < .001), but this difference was larger in the varied-mapping group (130 ms) than in the consistent-mapping group (93 ms; Group by Trial Type: *p* < .001). Thus, the varied-mapping group was more influenced by the presentation of invalid signals than the consistent-mapping group. This could be due to increased memory demands in the varied-mapping group, rule conflict/inertia caused by the frequent switching between signal-validity mappings (similar to task-set conflict/inertia in task switching; for reviews, see Kiesel et al., 2010; Monsell, 2003; Vandierendonck, Liefooghe, & Verbruggen, 2010), or both. However, the group differences were more pronounced in Experiments 1 and 2 than in Experiments 3 and 4 (Tables 1 and 4). As discussed above, we expected memory demands to be lower but switch demands to be higher in Experiments 3–4 than in Experiments 1–2. Therefore, the interaction with Experiment suggest that the larger interference effects in varied-mapping groups may be due difficulties with retrieving the relevant rule or cue from memory or difficulties with comparing the signal with the cue maintained in working memory (rather than switching per se).

RT distributions of no-signal and invalid-signal RTs should overlap when there is independence between go and stop. However, visual inspection of the group RT distributions shows the invalid-signal distribution was to the *right* of the no-signal distribution. Even the fastest go responses, which occurred approximately 150–200 ms after the presentation of the signal, were influenced by the presentation of invalid signals. This conclusion was further supported by a post hoc ANOVA that contrasted the RTs at the 10th percentile for no-signal and invalid signals trials for each group. There was a main effect of trial type in both mapping groups (*p*’s < .001 after correction for multiple comparisons). This indicates that in both groups, the fastest RTs were influenced by the presentation of invalid signals. In selective stop tasks and stop–change tasks, SSRT for healthy and young adults is usually around 250–350 ms (e.g. Bissett & Logan, 2014; Boecker et al., 2013; De Jong et al., 1995; Logan & Burkell, 1986; Verbruggen, Logan, Liefooghe, & Vandierendonck, 2008). Thus, this difference between the fastest no-signal and invalid-signal trials argues against independence between go and stop in *both* mapping groups, but the signal–respond data and the larger interference effects in the varied-mapping group indicate that this dependence between go and stop increases when the demands on the rule-based system increase.

The accuracy data were consistent with the RT data: Subjects made more errors on invalid-signal trials than on no-signal trials (Trial Type: *p* < .001), but this effect was larger in the varied-mapping group (7%) than in the consistent-mapping group (4%; Group by Trial Type: *p* < .001). A closer inspection of the error data (not shown) indicate the higher error rate on invalid-signal trials was primarily due to erroneous responses to the location of invalid signal (i.e. left/right responses). Tables 1 and 4 show that the accuracy difference between invalid-signal and no-signal trials was generally larger in Experiments 1 and 2 than in Experiments 3 and 4, but the Group by Trial Type interaction was not influenced by Experiment.

#### 2.3.3. Individual strategies

So far, we have assumed that all subjects use a ‘Discriminate then Stop’ strategy to perform the selective stop–change task (i.e. they first select the appropriate action when a signal occurs; they stop if the signal is valid, but they complete the go process if the signal is invalid). However, some researchers have argued that subjects can also use a ‘Stop then Discriminate’ strategy to perform selective stop tasks (for a review, see Bissett & Logan, 2014). When subjects use this strategy, they inhibit the response whenever a signal occurs^3^, and then they select the appropriate action: if the signal is valid, no further action is required; if the signal is invalid, they restart or re-execute the canceled go response. When the ‘Stop then Discriminate’ strategy is used, the context independence assumption of the independent race model is less likely to be violated because the decision about the validity of the signal is made after the response has been stopped (Bissett & Logan, 2014).

**Fig. 4:**
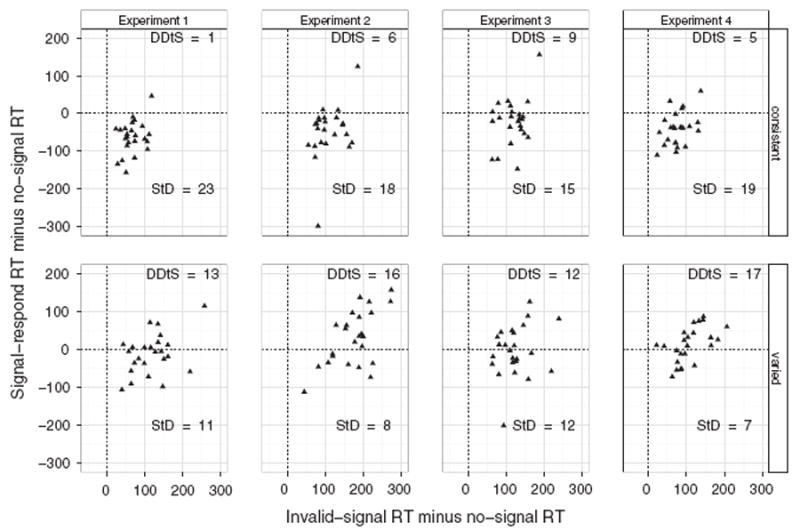
Difference scores for all subjects in the consistent-mapping and varied-mapping group for each experiment. The numbers in the graph indicate the number of subjects per strategy. DDtS = ‘Discriminate then Stop’ strategy, with dependence between go and stop; StD = ‘Stop then Discriminate’ strategy (see main text for further details).

We categorized each subject’s strategy using the decision matrix discussed in Bissett and Logan (2014, p. 457): If subjects use the ‘Stop then Discriminate’ strategy, signal–respond RT should be shorter than no-signal RT, but invalid-signal RT should be longer than no-signal RT (because subjects have to restart the response on invalid-signal trials). If subjects use a ‘Discriminate then Stop’ strategy, and the decision to stop or not interferes with going (i.e. dependence between go and stop), both signal–respond RT and invalid-signal RT should be longer than no-signal RT. If they use the ‘Discriminate then Stop’ strategy, and the decision to stop or not does not interfere with going (i.e. independence between go and stop), signal–respond RT should be shorter than no-signal RT, and no-signal RT and invalid-signal RT should be comparable. To determine whether signal–respond and invalid-signal RTs were longer than no-signal RT, we calculated for each subject the 95% confidence interval around their mean no-signal RT; their signal–respond and invalid-signal RTs were considered to be different from their no-signal RT if the signal RTs did not fall within this confidence interval. The outcome of this analysis appears in Fig. 4.

Most subjects in the consistent-mapping condition (in which the discrimination was easiest) seemingly used the ‘Stop then Discriminate’ strategy, whereas most subjects in the varied-mapping condition (in which the signal discrimination was hardest) seemingly used the ‘Discriminate then Stop’ strategy (Fig. 4). We observed this pattern of results in each experiment. When we combined the data of all experiments, Fisher’s Exact Test for Count Data revealed that the distribution of strategies across the varied-mapping and consistent-mapping groups was significantly different (*p* < .001).

From a strategy point of view, this pattern of results seems very odd. In the varied-mapping group, the signal-validity mapping constantly changed, so the demands on the rule-based system remained high throughout the whole experiment. Presumably, this should have encouraged subjects in this group to use a ‘Stop then Discriminate’ strategy rather than a ‘Discriminate then Stop’ strategy. After all, ‘Stop then Discriminate’ allows fast stopping and reduces overlap between demands of the stop and the go task on the rule-based system. Reducing the demands should be more important when they are high, so the varied-mapping group should prefer the ‘Stop then Discriminate’ strategy more than the consistent-mapping group. We found the opposite.

## 3. General discussion

Performance in various response inhibition tasks is usually described as an independent race between a go process and a stop process. In the past three decades, several studies have indicated that go and stop processing are indeed independent for most of their durations. However, dependency between going and stopping may arise when subjects are instructed to stop their response for some signals but not for others. The present study used a selective stop–change task with a consistent vs. varied-mapping manipulation to test whether decision difficulty influenced dependence between going and stopping.

### 3.1. Selective stop–change performance in the consistent and varied-mapping conditions

The analysis of the data of four experiments showed that mean signal–respond RT was shorter than mean no-signal RT in a consistent-mapping condition, but not in a varied-mapping condition. The presentation of invalid signals also slowed go processing, especially in the varied-mapping group. Furthermore, inspection of the RT distributions indicated that even the fastest responses were influenced by the presentation of signals. Based on SSRTs of previous studies, we estimate that interference between go and stop processing occurred well before the stop process was finished. Combined, these findings indicate that the decision to stop or not interfered with go processing, especially when the signal mapping varied. These findings challenge the independent race models for selective stopping.

Our findings also shed a new light on strategy use in selective stop tasks. We categorized each subject’s strategy using the decision matrix proposed in Bissett and Logan (2014; p. 457). Most subjects in the varied-mapping group seemingly used a ‘Discriminate then Stop’ strategy, with strong dependence between going and stopping, whereas most subjects in the consistent-mapping group seemingly used a ‘Stop then Discriminate’ strategy. We had expected the opposite pattern of results.

### 3.2. Capacity sharing in selective stop tasks

The main finding of our combined analysis is the dependence between going and stopping, especially in the varied-mapping condition (but note that inspection of the individual data also showed dependence for some subjects in the consistent-mapping group; Fig. 4). We propose that the discrimination or decision component of the selective stop–change task interferes with ongoing go processing in the primary task, and when stop difficulty increases, dependency increases. This effect may be similar to the dual-task costs observed in the psychological refractory period (PRP) paradigm.

In the PRP paradigm (Pashler, 1994; Telford, 1931; Welford, 1952), two stimuli are presented in rapid succession and subjects are instructed to respond to each stimulus as quickly as possible. The common finding is that responding to the second stimulus is delayed when the delay between the first and second stimulus is short, whereas responding to the first stimulus is usually not influenced much by the delay (for a short review, see Marois & Ivanoff, 2005). Dominant accounts of dual-task performance propose that selecting the response to the second stimulus does not start before response selection in the first task is finished (e.g. Logan & Gordon, 2001; Meyer & Kieras, 1997; Pashler, 1994; Pashler & Johnston, 1998). However, performance in the first task can be influenced by the selection of the second response (e.g. Hommel, 1998; Logan & Delheimer, 2001; Watter & Logan, 2006). This has led some researchers to propose capacity-sharing models of dual-task performance (e.g. Miller, Ulrich, & Rolke, 2009; Navon & Miller, 2002; Tombu & Jolicoeur, 2003). These capacity sharing accounts postulate that parallel processing can occur, but Task 1 and Task 2 have to share limited processing capacity. When Task 1 is prioritized (explicitly or implicitly), most processing capacity will be allocated to this task; consequently, response selection in Task 1 processing will not be influenced much by the presentation of the second stimulus, whereas response selection in Task 2 can only start properly when the response to the first stimulus has been selected. But when the tasks are prioritized more equally, responding in both tasks will be influenced (e.g. Miller et al., 2009).

**Fig. 5:**
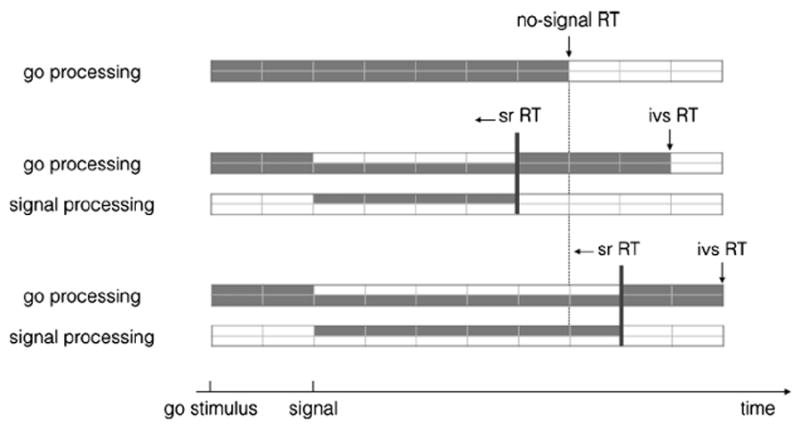
A schematic representation of ‘capacity sharing’ between go processing in the primary task and signal processing. The top panel depicts go processing on no-signal trials; the middle panel depicts go and signal processing in the consistent-mapping group; and the bottom panel depicts go and signal processing in the varied-mapping group. Go processing is triggered by the presentation if the go stimulus; signal processing is triggered by the presentation of the signal. On valid-signal trials, the primary-task response is inhibited when the stop process finished. For simplicity, we did not depict the execution components of the change response. RT = reaction time; sr RT = signal–respond RT; ivs RT = invalid-signal RT.

Based on the PRP literature, we propose a capacity sharing account for performance in selective stop tasks. This is shown in Fig. 5. The top panel of this figure depicts go processing on no-signal trials; the middle panel depicts go and signal processing on signal trials in the consistent-mapping group; and the bottom panel depicts go and signal processing on signal trials in the varied-mapping group. We assume that the go and signal processes will interact for their whole duration when a signal is presented. Furthermore, we assume that processing the signals in the varied-mapping condition is harder than in the consistent-mapping condition (for the reasons discussed above). Consequently, the decision to stop or not will finish later in the varied-mapping condition than in the consistent-mapping condition (indicated by the thick vertical lines in Fig. 5), and go and stop processing will interact for a longer period. This can easily explain the RT differences between conditions. Fig. 5 shows that signal–respond RT will be shorter than no-signal RT when the decision is easier (middle panel), whereas it will be longer than no-signal RT when the decision to stop or not is difficult (bottom panel). The figure also shows that the interaction between going and stopping will cause invalid-signal RT to be longer than no-signal RT, but this difference will be more pronounced in the varied-mapping condition than in the consistent-mapping condition. In other words, we do not have to assume that subjects in the varied-mapping condition used a categorically different strategy than subjects in the consistent-mapping condition. Our limited capacity sharing account can explain the RT patterns in both groups. Note that we have depicted stop-signal processing by a single bar, but we assume that many processes contribute to successfully stopping a response (see Section 3.5). Here we propose that response selection in the go task has to share capacity the decision to stop or not, which could involve retrieval or activation of the relevant signal rule, a comparison of the signal with the cue, conjunctive feature evaluation, or a combination of these processes. When the signal is considered to be valid, a neural inhibitory process will be activated. Interactive race models have shown that at this point, go and stop will also briefly interact.

We have previously formalized the concept of ‘processing capacity’ as a measure of the rate of processing (Logan et al., 2014): A process has unlimited capacity if its rate is unchanged when another process enters the race, whereas it has limited capacity if its rate decreases as more runners enter the race (see also Bundesen, 1990; Logan & Gordon, 2001; Townsend & Ashby, 1983). We developed a special independent race model that describes the race between going and stopping as stochastic accumulators, and examined versions in which the go process and stop process shared capacity and did not share capacity. When we manipulated the difficulty of the go task and occasionally presented a single signal we found that the stop rate parameter was not influenced. This indicates that stopping did not share capacity with going in a standard stop task (Logan et al., 2014). Versions of the model in which going and stopping share capacity might fit the results of the present study better, so a future goal of our research program is to fit our diffusion race model to the present data.

Capacity as measure of processing rate describes the consequences, but it does not necessarily describe why processing rates decrease when extra choice alternatives are added or when other processes enter the race. We hypothesize that limited capacity arises from competition between representations. Biased competition accounts of visual attention assume that visual processing is competitive: the stronger the response to a particular object, the weaker the response to other objects (e.g. Beck & Kastner, 2009; Bundesen, 1990; Desimone & Duncan, 1995; Duncan, 2006; Kastner & Ungerleider, 2000). Thus, when extra stimuli are added, processing rates for the other stimuli will decrease. This competition can be biased in a top-down fashion, allowing people to focus on task-relevant information. In a similar vein, many models of action selection assume that multiple action options will compete, so that support for one option reduces the (relative) support for the alternatives (e.g. Cisek & Kalaska, 2010; Logan & Gordon, 2001; Usher & McClelland, 2001). Again, this competition can be biased (e.g. Logan & Gordon, 2001). More generally, competition between representations has been used to account for limitations in working memory capacity (e.g. Oberauer, 2009), and the broader difficulty of doing several things at once (Duncan, 2006). In sum, the biased competition idea seems to provide a general description of how the cognitive and neural system processes information, and for why concurrent processes sometimes appear to share limited capacity (but see e.g. Navon & Miller, 2002).

### 3.3. Simple stopping as a prepared reflex?

In selective stop tasks (including our selective stop–change task), ongoing processes interfere with each other. But several studies indicate that in the stop-signal task and stop–change tasks in which all signals are valid, the stop process does not interfere with ongoing go processing (except for a very brief period of interaction near the end of SSRT; Boucher et al., 2007; Logan et al., 2015). For example, manipulating the difficulty of the response-selection processes in the go task does not influence stopping performance when all signals are valid (Logan, 1981; Logan et al., 1984, 2014). Other studies showed that stopping in a standard stop-signal task or stop–change task does not suffer from dual-task interference (Hübner & Druey, 2006; Logan & Burkell, 1986; Yamaguchi et al., 2012). So why did we observe strong dependence between going and stopping (violating the context independence assumption of the independent race model)?

Consistent with standard PRP models, we assume that various forms of action control, including stopping, rely on signal detection, selection of an appropriate action, and the activation of the stop response or stopping network (Verbruggen, McLaren, & Chambers, 2014). In selective stop tasks, the selection can be quite difficult, especially when the signal mapping constantly changes. By contrast, in stop-signal and stop–change tasks with only one signal, selection of the appropriate action is very straightforward. Consequently, stopping will not interfere much with the primary task.

The idea that selection demands are low in standard stop-signal and stop–change tasks is also consistent with the idea that most of SSRT in these tasks is occupied by afferent or sensory processes (Boucher et al., 2007; Logan et al., 2014, 2015; Salinas & Stanford, 2013). One could even speculate that stopping in standard stop-signal tasks is a ‘prepared reflex’ (e.g. Hommel, 2000; Logan, 1978; Meiran, Cole, & Braver, 2012). Several studies indicate that goal-directed actions may not require much control anymore once the task instructions are properly implemented: ‘the components of the task seem automatic, but the task itself is not’ (Logan, 1978, p. 57). In stop tasks with only one signal, stopping could be a prepared reflex due to the low signal selection demands. Once the task instructions are implemented (‘IF signal THEN stop’), the stop process can be triggered easily by the presentation of the stop signal; consequently, stop processing and rule-based (or algorithmic) primary-task processing can occur in parallel without much dual-task interference (cf. Kahneman, 2003; Logan, 1979, 1988; Schneider & Shiffrin, 1977; Shiffrin & Schneider, 1977).

We should point that capacity sharing can occur in stop-signal tasks with a single stop signal. The stop rate parameters depend on the discriminability, intensity, and modality of the stop signal (e.g. van der Schoot, Licht, Horsley, & Sergeant, 2005), which could be interpreted as a capacity limitation (Logan et al., 2014). Furthermore, we have recently demonstrated that competition between visual signals in the go and the stop tasks influences stopping (Verbruggen, Stevens, & Chambers, 2014), which is consistent with the idea that stimuli have to compete for limited processing capacity. Thus, it seems that under certain circumstances, capacity sharing may also occur in simple stop-signal tasks.

### 3.4. Categorically different stopping strategies? Maybe not

Our results are very similar to those observed in previous selective stop studies. As discussed above, two main selective stopping strategies have been proposed in this literature: the ‘Stop then Discriminate’ strategy (also know as the ‘Stop-Restart’ strategy; e.g. Aron, Behrens, Smith, Frank, & Poldrack, 2007) and the ‘Discriminate then Stop’ strategy. To distinguish between these two strategies, researchers have relied on differences between no-signal RTs, signal–respond RTs, and invalid-signal RTs. But our analysis indicates that such RT differences could be due to capacity sharing and the difficulty of the discrimination process. In other words, our study indicates that RT differences between the groups, subjects, or conditions in selective stop tasks could be quantitative (i.e. degree of capacity sharing) rather than qualitative (i.e. different strategies).

This is not to say that strategies have no role in selective stop tasks. Many studies indicate that people use various strategies to control their actions (for reviews, see e.g. Aron, 2011; Braver, 2012). For example, subjects can ‘proactively’ adjust attentional and response-selection parameters in the go task to enhance stop-signal detection and slow down responding (e.g. Aron, 2011; Verbruggen & Logan, 2009b; Verbruggen, Stevens, et al., 2014). Furthermore, task prioritization or task bias can be a top-down strategic decision (e.g. Logan & Gordon, 2001; Miller et al., 2009). For example, it may be advantageous to prioritize the stop process (and allocate more processing capacity to it) when signals are likely to be valid (see Bissett & Logan, 2014).

### 3.5. Implications and practical guidelines for stop-signal users

In the present study, we found strong dependence between stopping and going in selective stopping tasks, and we have argued that capacity sharing may also occur in stop-signal tasks. In other words, the present study and other recent work indicates that going and stopping tend to interact when stopping is no longer a ‘simple’ prepared reflex. Consequently, the independence assumptions of the independent race model will be violated. As discussed in the Introduction, the assumptions should not be taken lightly because SSRT cannot be reliably estimated when they are violated. Therefore, we propose that every stop-signal study that uses the tracking procedure and estimates SSRT should:

1. Report average signal–respond RT, and confirm that it is statistically different from average no-signal RT for each experimental condition.
2. Determine whether differences between conditions indicate various degrees of capacity sharing.
3. Confirm that signal–respond RT is shorter than no-signal RT for every subject for whom SSRT is estimated. SSRT should not be estimated for subjects with signal–respond RTs longer than no-signal RTs. The number of subjects excluded from the SSRT analysis should be mentioned.

These first three guidelines focus on testing the independence assumptions. In addition, stop-signal users should always report:

1. The probability of responding on signal trials for each condition
2. The average stop-signal delay for each condition
3. Use an appropriate method, like the integration method, to estimate SSRT (Verbruggen et al., 2013).

A final note concerns the interpretation of the SSRT. SSRT measures the time it takes to stop a response. However, it is important to realize that SSRT is a global concept that describes the chain of processes involved in an act of control that results in a response being withheld (Verbruggen, McLaren, et al., 2014). More specifically, SSRT captures the duration of perceptual, decisional, and (inhibitory) motor-related processes. For example, previous behavioral studies and computational work have highlighted the role of perceptual processes (see above). Our study shows that successfully stopping also depends on decisional processes, such as response selection and memory retrieval (see also e.g. Logan et al., 2014; van de Laar, van den Wildenberg, van Boxtel, & van der Molen, 2010). Finally, when the decision to stop is reached, motor output or other ongoing processing has to be suppressed (e.g. via a fronto-basal-ganglia network). Thus, in simple stop-signal tasks and their many variants, SSRT reflects more than the duration of a single neural inhibitory process, and researchers should consider at which processing stage(s) differences between groups or conditions arise (for a more elaborate discussion of this issue, see e.g. Verbruggen, McLaren, et al., 2014).

## 4. Conclusion

Almost every stop-signal paper assumes that going and stopping occur independently. Many papers have provided direct empirical and computational support for this assumption. Violations of the independence assumption have important theoretical implications, and practical implications for the estimation of the stopping latency. In the present study, we found dependence between stopping and going in selective stop tasks, especially when signal discrimination was difficult. We propose that in selective stop tasks, the decision to stop or not will share limited processing capacity with the go task. The limited processing capacity arises from competition between go and stop representations. The capacity sharing idea can account for performance differences between groups, subjects, and conditions. For example, when the decision is difficult, the go and stop task will have to share capacity for a longer period, resulting in longer RTs on signal trials. Our account can also explain why the go and stop tasks are largely independent in simple stop-signal tasks, since the decision to stop or not when a signal occurs is very simple in these tasks.

## Supplementary Material

1

## Figures and Tables

**Table 1:** Overview of go performance on no-signal trials and invalid-signal trials: probability of an accurate go response [*p*(correct)] and average reaction time (RT) for correct go responses as a function of Group (consistent-mapping vs. varied-mapping), Experiment, and Trial Type (no signal vs. invalid signal).

	Experiment 1		Experiment 2		Experiment 3		Experiment 4	
	*M*	*sd*	*M*	*sd*	*M*	*sd*	*M*	*sd*
*P(correct)*								
Consistent mapping								
No signal	0.986	0.009	0.962	0.019	0.965	0.022	0.974	0.029
IV signal	0.944	0.074	0.899	0.070	0.926	0.037	0.954	0.052
Varied mapping								
No signal	0.976	0.021	0.946	0.035	0.978	0.016	0.980	0.018
IV signal	0.913	0.060	0.822	0.084	0.918	0.050	0.940	0.048
*Go RT*								
Consistent mapping								
No signal	664	165	726	131	569	108	597	115
IV signal	730	155	833	134	687	111	671	121
Varied mapping								
No signal	695	122	709	110	611	120	624	110
IV signal	807	131	883	104	734	121	731	130

**Table 2:** Overview of performance on valid-signal trials: Probability of responding on a valid-signal trial [*p*(respond|signal)], average valid change-signal delay (CSD), average reaction time for go responses on signal–respond trials (signal–respond RT), the difference between signal–respond RT and no-signal RT (both correct and incorrect responses were included when mean no-signal RT was calculated), and average reaction time for the change response (Change RT), as a function of Group (consistent-mapping vs. varied-mapping) and Experiment. Change RT corresponds to the time interval between the presentation of the valid signal and the left/right key press.

	*p*(respond|signal)		CSD		Signal–respond RT		No-signal RT minus signal–respond RT		Change RT	
	*M*	*sd*	*M*	*sd*	*M*	*sd*	*M*	*sd*	*M*	*sd*
*Experiment 1*										
CM	0.480	0.069	352	172	601	151	−63	44	597	97
VM	0.472	0.081	348	145	682	117	−12	54	649	84
*Experiment 2*										
CM	0.461	0.070	353	134	679	115	−48	72	696	85
VM	0.479	0.076	282	129	741	109	30	73	797	108
*Experiment 3*										
CM	0.490	0.061	253	103	546	86	−22	61	682	101
VM	0.503	0.083	272	126	606	109	−4	68	667	84
*Experiment 4*										
CM	0.490	0.058	279	122	557	97	−41	43	620	64
VM	0.490	0.073	275	124	635	117	12	46	659	137

Note: Change RT was higher in the varied-mapping condition than in the consistent-mapping condition in Experiments 1–2 (both *p*’s < .001), but the group differences were not statistically significant in Experiments 3 and 4, *p* = .63 and *p* = .20, respectively.

**Table 3:** Performance on signal–respond trials was analyzed by means of mixed ANOVAs with Group (consistent-mapping or varied-mapping) and Experiment as a between-subjects factors and Trial Type (no-signal vs. signal–respond) as within-subjects factor.

	*Df*1	*Df*2	*SS*1	*SS*2	*F*	*p*	*p* < .05	*η*^2^*_gen_*
Experiment	3	184	1,022,371	4,900,577	12.796	0.000	*	0.164
Condition	1	184	197,333	4,900,577	7.409	0.007	*	0.036
Trial Type	1	184	32,495	318,397	18.779	0.000	*	0.006
Experiment by Condition	3	184	16,487	4,900,577	0.206	0.892		0.003
Experiment by Trial Type	3	184	12,169	318,397	2.344	0.074		0.002
Condition by Trial Type	1	184	59,959	318,397	34.650	0.000	*	0.011
Experiment:Condition:Trial Type	3	184	10,813	318,397	2.083	0.104		0.002

**Table 4:** Overview of the Analyses of Variance of no-signal and invalid-signal trials. Performance was analyzed by means of mixed ANOVAs with Group (consistent-mapping or varied-mapping) and Experiment as a between-subjects factor and Trial Type (no-signal vs. invalid-signal) as within-subjects factor.

	*Df*1	*Df*2	*SS*1	*SS*2	*F*	*p*	*p* < .05	*η*^2^*_gen_*
*p(correct)*								
Experiment	3	184	0.172	0.555	18.998	0.000	*	0.180
Condition	1	184	0.029	0.555	9.714	0.002	*	0.036
Trial Type	1	184	0.305	0.226	248.4	0.000	*	0.281
Experiment by Condition	3	184	0.035	0.555	3.858	0.010	*	0.043
Experiment by Trial Type	3	184	0.052	0.226	13.995	0.000	*	0.062
Condition by Trial Type	1	184	0.022	0.226	18.170	0.000	*	0.028
Experiment:Condition:Trial Type	3	184	0.007	0.226	2.007	0.115		0.009
*RT*								
Experiment	3	184	1,208,409	5,609,615	13.212	0.000	*	0.173
Condition	1	184	149,897	5,609,615	4.917	0.028	*	0.025
Trial Type	1	184	1,165,417	162,309	1321.2	0.000	*	0.168
Experiment by Condition	3	184	19,017	5,609,615	0.208	0.891		0.003
Experiment by Trial Type	3	184	44,102	162,309	16.665	0.000	*	0.008
Condition by Trial Type	1	184	34,192	162,309	38.762	0.000	*	0.006
Experiment:Condition:Trial Type	3	184	12,069	162,309	4.561	0.004	*	0.002

## Footnotes

1 Exploratory analyses (not shown) indicated that subjects in both groups of Experiment 1 were more distracted by invalid signals that had the same color as the valid signal (compared with shape overlap or no overlap). This difference between color and shape could have been due to the order of the words in the cue (i.e. color–shape), or the relative salience of the features. To rule out the first possibility, we counterbalanced feature order in the cues of Experiment 2.

2 Wessel and Aron (2014) found that the more features stimuli shared with the valid signal, the more they slowed responding. We replicated this finding (see Supplementary Materials).

3 Responses on invalid-signal trials could also be stopped in a bottom-up fashion. Several studies have indicated that stopping can be primed by stimuli or stimulus features that were previously associated with stopping (Bissett & Logan, 2011; Giesen & Rothermund, 2014; Rieger & Gauggel, 1999; Verbruggen & Logan, 2008a,b, Verbruggen, Best, Bowditch, Stevens, & McLaren, 2014; Verbruggen, Logan, et al., 2008).
