# Supplementary material for: Evidence for capacity sharing when stopping
Source: Cognition. Author manuscript; Available in PMC 2016 Apr 8. (PMC4787292; doi:10.1016/j.cognition.2015.05.014)
Supplement: 1 [file NIHMS753226-supplement-1.rtf]

Supplementary File
Wessel and Aron (2014) recently found that stimuli that partially overlapped with the relevant stop signal slowed down responding; time-frequency analyses of the electroencephalogram (EEG) suggest that this slowing was associated with activation of a neural network that was also activated on valid-signal trials. 
	To further explore the effect of feature overlap on go performance in our study, we compared invalid signals that shared a feature with the valid signal (e.g. a blue square when the valid signal was a red square) with invalid signals that did not share a feature with the valid signal (e.g. a blue square when the valid signal was a red circle). Descriptive statistics appear in Table S1; inferential statistics appear in Table S2. Subjects in both groups responded slower to the go stimulus (25 ms; Trial Type: p < .001) on invalid-signal trials with a feature overlap compared with invalid-signal trials without feature overlap. This feature-overlap effect did not interact with Experiment or Condition. 
	Wessel and Aron (2014) concluded that stimuli that partially overlapped with the relevant stop signal could activate a braking or stopping network. However, this braking account cannot easily explain why signal-respond RT is sometimes longer than no-signal RT in selective stop tasks. Therefore, we propose a capacity-sharing account in the General Discussion of the main manuscript. This account provides the most parsimonious explanation for the overall RT pattern observed in the present study. 

Table S1. Average reaction time (RT) for correct go responses as a function of Group (consistent-mapping vs. varied-mapping), Experiment, and Trial Type (no-overlap vs. feature-overlap).

		Experiment 1	Experiment 2	Experiment 3	Experiment 4	
		M	sd	M	sd	M	sd	M	sd	
Consistent Mapping									
	No Signal	710	159	812	125	672	109	655	125	
	IV Signal	740	155	844	141	695	115	679	121	
Varied Mapping									
	No Signal	786	132	875	102	719	117	716	133	
	IV Signal	818	131	887	109	742	126	739	131	

Table S2: Performance was analyzed by means of mixed ANOVAs with Group (consistent-mapping or varied-mapping) and Experiment as a between-subjects factor and Trial Type (no overlap vs. feature-overlap) as within-subjects factor. 

	Df1	Df2	SS1	SS2	F	p	p < .05	η2gen	
	Experiment	3	184	1494909	5869581	15.621	0.000	*	0.199	
	Condition	1	184	337837	5869581	10.591	0.001	*	0.053	
	Trial Type	1	184	59274	141788	76.921	0.000	*	0.010	
	Experiment by Condition	3	184	12481	5869581	0.130	0.942		0.002	
	Experiment by Trial Type	3	184	1202	141788	0.520	0.669		0.000	
	Condition by Trial Type	1	184	446	141788	0.579	0.448		0.000	
	Experiment:Condition:Trial Type	3	184	1897	141788	0.820	0.484		0.000	
